# Taxonomic study on specimens of the genus *Micrencaustes* deposited in the Bernice P. Bishop Museum (Coleoptera, Erotylidae)

**DOI:** 10.3897/zookeys.645.11003

**Published:** 2017-01-12

**Authors:** Jing Li, Yan-Chen Zhao, Guo-Dong Ren, Zhiqiang Cheng

**Affiliations:** 1 College of Plant Protection, Agricultural University of Hebei, Baoding, Hebei, 071002, P. R. China; 2 College of Life Sciences, Hebei University, Baoding, Hebei 071002, P. R. China; 3 Department of Plant and Environmental Protection Sciences, CTAHR, University of Hawaii at Manoa, Honolulu, HI, 96822, USA

**Keywords:** Philippines, Australia, key, new species, taxonomy

## Abstract

Specimens of the genus *Micrencaustes* deposited in Bernice P. Bishop Museum were studied. Two new species Micrencaustes (Mimencaustes) rotundimaculata **sp. n.** and Micrencaustes (Mimencaustes) serratimaculata **sp. n.** are described and illustrated. A key to worldwide species of the subgenus Mimencaustes is provided.

## Introduction

The genus *Micrencaustes* was established by Crotch (1876) for *Encaustes lunulata* (MacLeay, 1825). This genus includes two subgenera and 42 species, distributed mainly in Asia and Australia (Heller 1918; Chûjô and Chûjô 1989). It is characterized by small antennal clubs, coarse eyes, a very broad terminal segment of the maxillary palpus, and possession of procoxal lines. The subgenus Mimencaustes Heller has mesocoxal lines, whereas subgenus Micrencaustes does not. Recent taxonomic work on the genus includes Osawa and Chûjô (1990), Li and Ren (2006) and Meng et al. (2014).

In the past, M. Chûjô (1968a, b) examined specimens of Erotylidae in the Bernice P. Bishop Museum (BPBM), Honolulu, Hawaii, U.S.A. He mainly studied specimens from Thailand, Laos, Vietnam, and southern China. He mentioned only one species of the genus *Micrencaustes*, *Micrencaustes liturata* MacLeay. In 2016, the first author researched the specimens of *Micrencaustes* in BPBM, and this paper presents the result of this study. The material examined included 108 specimens representing 15 species. Among them, seven and eight species belonged to the subgenera *Micrencaustes* and *Mimencaustes*, respectively. Two new species of the subgenus Mimencaustes were described and illustrated. One new species, Micrencaustes (Mimencaustes) rotundimaculata sp. n., was collected from Philippines. The other new species, Micrencaustes (Mimencaustes) serratimaculata sp. n., was collected from Australia.

## Materials and methods

Morphological examinations were carried out with a Nikon SMZ1500 stereomicroscope. To examine the genitalia, the abdominal segments were detached from the body after softening in hot water. All measurements are given in millimeters. Holotypes and a paratype are deposited in BPBM. Morphological terminology follows Wegrzynowicz (1997) and Skelley and Leschen (2007). The following abbreviations are used in the text: pl, pronotum length; pw, pronotum width.

## Taxonomy

### Key to the worldwide species of the subgenus Mimencaustes Heller

**Table:** 

1	Body entire dark, without marks	**2**
–	Body with marks	**3**
2	Body strongly shining	**Micrencaustes (Mimencaustes) papuana Heller**
–	Body weakly shining	**Micrencaustes (Mimencaustes) dehaanii (Castelnau)**
3	The marks on pronotum and elytron	**4**
–	The marks only on pronotum or elytron	**6**
4	Pronotum without black spots in the mark	**Micrencaustes (Mimencaustes) serratimaculata sp. n.**
–	Pronotum with one or two black spots in the mark	**5**
5	Head with an irregular red mark between eyes, antennomere III almost equal to antennomere IV	**Micrencaustes (Mimencaustes) torquata Gorham**
–	Head without mark between eyes, antennomere III approx 1.5 times as long as antennomere IV	**Micrencaustes (Mimencaustes) taiwana Araki**
6	Pronotum with marks	**7**
–	Elytron with marks	**8**
7	Head without orange mark, prosternal femoral lines surpassing the front edge of coxae	**Micrencaustes (Mimencaustes) acridentata Li & Ren**
–	Head with orange mark, prosternal femoral lines reaching the front edge of coxae	**Micrencaustes (Mimencaustes) renshiae Meng, Ren & Li**
8	Basal mark of elytron with two black spots near anterior border	**9**
–	Basal mark of elytron without black spots near anterior border	**10**
9	Pronotum with impunctate longitudinal median areas; every tibia with outer edge of apex acutely toothed	**Micrencaustes (Mimencaustes) michioi Osawa**
–	Pronotum without impunctate longitudinal median areas; mesotibia with outer edge of apex acutely toothed	**Micrencaustes (Mimencaustes) biomaculata Meng, Ren & Li**
10	Basal mark of elytron emarginated on posterior border	**Micrencaustes (Mimencaustes) wunderlichi Heller**
–	Basal mark of elytron not emarginated on posterior border	**11**
11	Elytron with posterior mark longitudinally oval	**Micrencaustes (Mimencaustes) dajaca Heller**
–	Elytron with posterior mark rounded	**Micrencaustes (Mimencaustes) rotundimaculata sp. n.**

#### Micrencaustes (Mimencaustes) rotundimaculata sp. n.

Taxon classificationAnimaliaColeopteraErotylidae

http://zoobank.org/08D1B989-6F0B-4388-ACB5-B223BBA52082

##### Type material.

Holotype. male, PHILIPPINES: Camarines Sur, Mt. Iriga, 13.4158°N, 123.4211°E, alt. 500-600m, 22 April 1962, H.M. Torrevillas leg. Paratype. 1 female, PHILIPPINES: Queznn, Queznn Park Tayahas[Note: The spelling on the label is wrong. Quezon, Quezon Park Tayabas is correct.], 14.6509°N, 121.0443°E, alt. 305m. 5 June 1932, F.C. Hadden leg.

##### Diagnosis.

Body elongated, widest at base of elytra, general color dark. Each elytron with two orange marks; anterior mark almost reaching lateral and basal margins, quadrate; the posterior one placed before the apex, rounded. Clypeus with the anterior border shaped like a concave “V”. Antennomere III approx. 1.4 times as long as IV; relative lengths of antennomeres II–XI: 11: 25: 18: 18: 18: 17: 16: 19: 12: 16. The terminal segment of maxillary palpus triangular, with sides rounded, nearly 2.9 times as wide as long. The elytron with strong striae, intervals finely and sparsely punctured. Mesoventrite with a median transverse rectangular depression.

##### Description.

**Figure 1.**
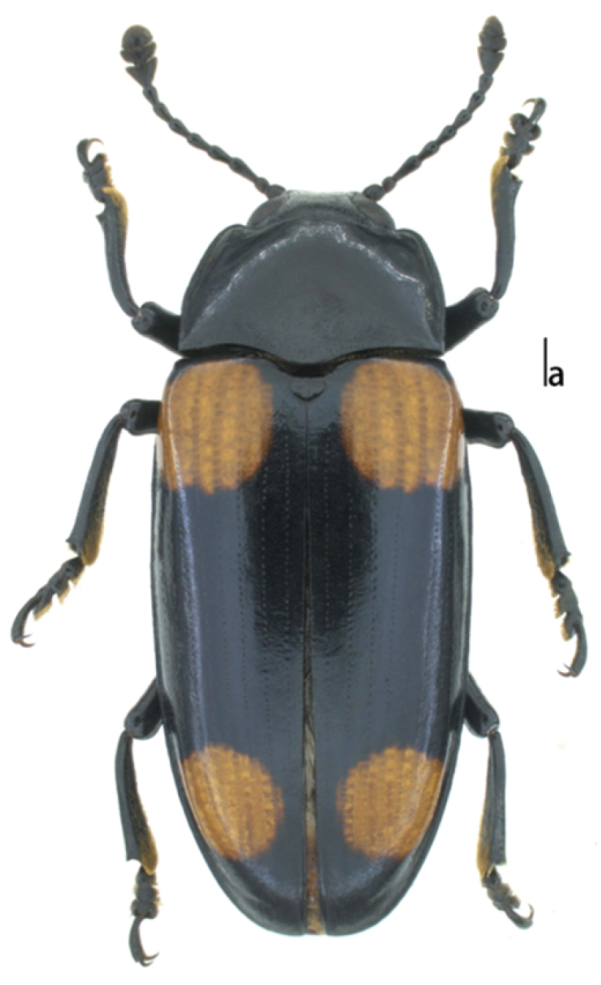
Habitus of Micrencaustes (Mimencaustes) rotundimaculata sp. n. Scale bar 1.0 mm.

*Body* (Fig. 1) elongate, moderately convex, length: 15.0–16.0mm, width: 5.0–5.2mm; widest at base of elytra, general color dark, moderately shining. Each elytron with two orange marks; anterior mark almost reaching lateral and basal margins, quadrate; the posterior one placed before the apex, rounded, not touching either margin.

**Figures 2–10.**
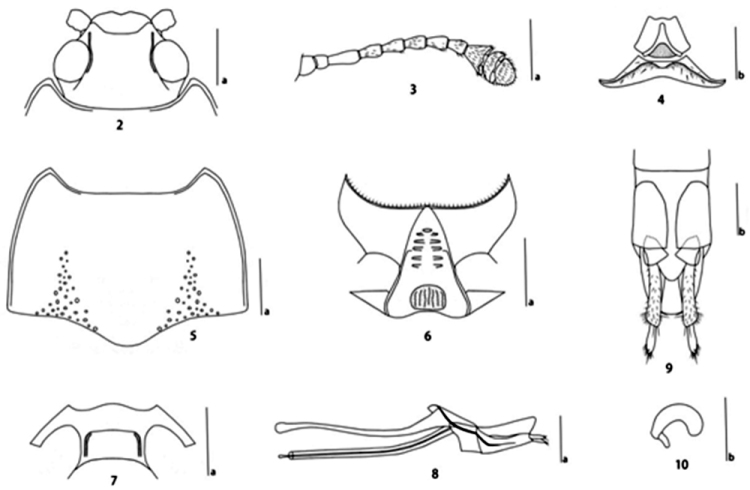
Micrencaustes (Mimencaustes) rotundimaculata sp. n. **2** head **3** antenna **4** mentum and submentum **5** pronotum **6** prosternum **7** mesoventrite **8** aedeagus in lateral views **9** female genitalia in ventral view **10** female spermatheca. Scale bars **a** 1.0 mm, **b** 0.5 mm.

*Head* (Fig. 2) strongly and sparsely punctured, densely punctured behind the eyes, with ocular lines. Clypeus strongly and rather densely punctured, with anterior border “V” concave, with a fovea on each side of the base. Eyes large, moderately prominent and coarsely faceted. Antennae (Fig. 3) extending to posterior border of pronotum; antennomere III nearly 1.4 times as long as IV; antennomere VIII slightly shorter than VII; antennomere IX triangular; antennomere X crescent-shaped; antennomere XI almost fan-shaped; relative lengths of antennomeres II–XI: 11: 25: 18: 18: 18: 17: 16: 19: 12: 16. The terminal segment of maxillary palpus triangular, with sides rounded, nearly 2.9 times as wide as long. Mentum (Fig. 4) triangular, with long golden setae, both sides concave; submentum (Fig. 4) depressed on each side of front area, without puncture, with long golden setae.

*Pronotum* (Fig. 5) widest at basal third (pl/pw = 0.76–0.77); sides almost parallel on posterior third, and slightly narrowing toward apex. Pronotum finely and densely punctured; with a group of coarse punctures on each side of base. Anterior angles projected; posterior angles nearly rectangular. Pronotal anterior margin concave and basal margin weakly sinuate.

*Prosternum* (Fig. 6) with shallow and oblique rugulae on lateral areas.

*Prosternal process* triangular, produced into a blunt point at apex, emarginate at posterior border, finely punctured at front, with a rounded depression in the middle of base, covered longitudinal wrinkles. Prosternal femoral lines almost straight, converging anteriorly and slightly exceeding the front edge of coxae.

*Scutellum* pentagonal, with fine and sparse punctures.

*Elytra* widest near base, then gradually narrowing to apex. Each elytron with nine striae, the outside stria short; strial punctures stronger at base, gradually weakened apically and disappearing before extremity; intervals finely punctured and wrinkled.

*Mesoventrite* (Fig. 7) broad, with a median transverse rectangular depression, coxal lines short, sternum with fine and sparse punctures.

*Metaventrite* finely and sparsely punctured, with a longitudinal depression in the middle of basal seven eighths.

*Abdomen* densely punctured, covered with short golden hairs.

*Male genitalia* (Fig. 8) with median lobe weakly curved, truncated at apex in lateral view; median strut 1.73 times as long as median lobe.

*Female genitalia* (Fig. 9) with narrow styli at apex of coxite, covered with setae at apex. Female spermatheca kidney-shaped (Fig. 10).

##### Distribution.

Philippines (Camarines Sur and Quezon).

##### Remarks.

Micrencaustes (Mimencaustes) rotundimaculata is most similar to Micrencaustes (Mimencaustes) dajaca Heller, 1918, due to the similar form and pattern of the elytron. The new species can be distinguished from it by: the elytron with an anterior quadrate mark, and a rounded posterior mark; clypeus with the anterior border shaped like a concave “V”; mesoventrite with a median transverse rectangular depression; and the elytron with strong striae, intervals finely and sparsely punctured. In contrast, Micrencaustes (Mimencaustes) dajaca has two elongate rounded marks on each elytron; clypeus with anterior border feebly emarginated; mesoventrite with a transverse arched depression in the middle; elytron with weak striae, intervals finely and densely punctured.

##### Etymology.

The species is named for having the posterior band of the elytron rounded.

#### Micrencaustes (Mimencaustes) serratimaculata sp. n.

Taxon classificationAnimaliaColeopteraErotylidae

http://zoobank.org/FECAA575-EF10-4F16-BD8D-99EAB4019A37

##### Type material.

Holotype. Female, Australia: Queensland, Hambledon [Note: Now named Edmonton], 17.0165°S, 145.7487°E, November 1921, Pemberton leg.

##### Diagnosis.

Body oblong oval, convex, general color dark, shiny. Pronotum with one transverse, zigzag dark red mark. Each elytron with two dark red bands. Clypeus anterior border feebly emarginated. Antennae short, antennomere III 1.7 times as long as IV; relative lengths of antennomeres II–XI: 22: 58: 34: 36: 34: 34: 30: 42: 27: 30. The terminal segment of maxillary palpus triangular, with rounded sides, nearly 2.9 times as wide as long. Pronotum widest at middle, with a group of coarse punctures on each side of base. Scutellum almost triangle, not transverse. Mesoventrite broad, with coxal lines. Abdomen finely and closely punctured. The last segment of abdomen covered with very large punctures along the outside edge. Mesotibia with outer edge of apex acutely toothed.

##### Description.

**Figure 11.**
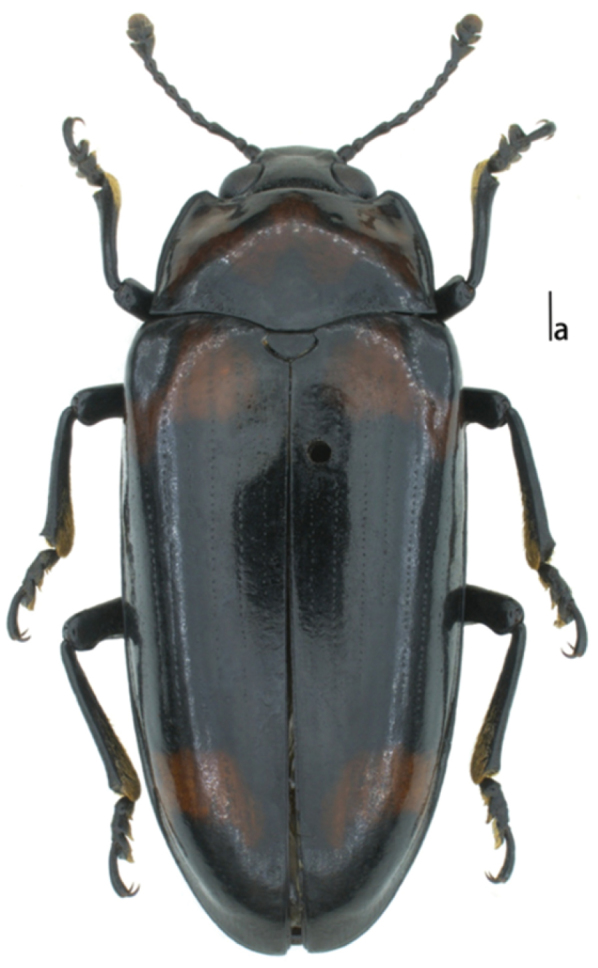
Habitus of Micrencaustes (Mimencaustes) serratimaculata sp. n. Scale bar 1.0 mm.

*Body* (Fig. 11) oblong oval, convex, length: 20.0 mm, width: 7.0 mm; general color dark, shining. Pronotum with one transverse, zigzag dark red mark occupying the sides and most of the center, with three waves at anterior border, with posterior border four waves. Each elytron with two dark red bands; anterior band at the base surrounding the humeral angle, leaving a black part at humerus; posterior band near the apex, neither touching the side nor suture, with posterior border curved.

**Figures 12–18.**
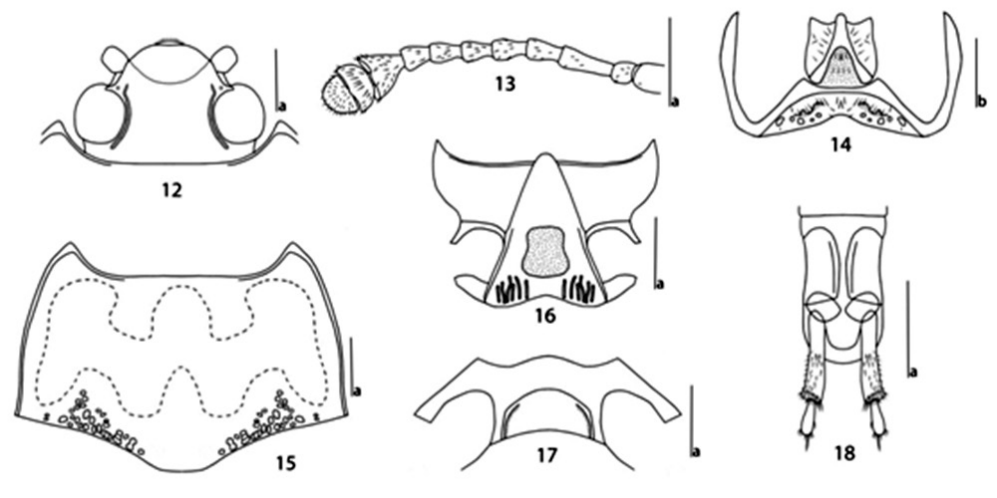
Micrencaustes (Mimencaustes) serratimaculata sp. n. **12** head **13** antenna **14** mentum and submentum **15** pronotum **16** prosternum **17** mesoventrite **18** female genitalia in ventral view. Scale bars **a** 1.0 mm, **b** 0.5 mm.

*Head* (Fig. 12) finely and sparsely punctured on vertex, strongly and sparsely punctured behind the vertex, with ocular lines. Clypeus strongly and sparsely punctured, with anterior border feebly emarginated, with a fovea on each side of base. Eyes large, moderately prominent laterally, coarsely faceted. Antennae (Fig. 13) short, approaching posterior border of pronotum; antennomere III 1.7 times as long as IV; antennomere VIII slightly shorter than VII; antennomere IX triangular; antennomere X crescent-shaped; antennomere XI semicircle; relative lengths of antennomeres II–XI: 22: 58: 34: 36: 34: 34: 30: 42: 27: 30. The maxillary palpus terminal segment triangular, sides rounded, nearly 2.9 times as wide as long. Mentum (Fig. 14) with plate triangular, both sides concave; submentum (Fig. 14) depressed on each side of middle area, with strong puncture and a few long golden setae.

*Pronotum* (Fig. 15) widest at middle (pl/pw = 0.71); sides slightly curved, strongly margined, with some coarse punctures on the surface; anterior margin straight in the middle, margined behind eyes; basal margin weakly sinuate. Pronotum finely and sparsely punctured; with a group of coarse punctures on each side of base. Anterior angles projected; posterior angles obtuse.

*Prosternum* (Fig. 16) sparsely punctured laterally, with some shallow and oblique rugulae; a depression in the middle area; surface with short golden setae.

*Prosternal process* triangular, produced into a blunt point at apex, emarginated on posterior border, sparsely punctured on point, with longitudinal rugulae at front. Prosternal femoral lines converging anteriorly and slightly exceeding to the front edge of coxae.

*Scutellum* almost triangle, posterior angle blunt, and surface with fine and sparse punctures.

*Elytra* widest near base, then gradually narrowing to apex. Each elytron with seven striae; intervals finely punctured and wrinkled.

*Mesoventrite* (Fig. 17) broad, almost no punctures, with a transverse arched depression medially, with coxal lines; sternum with fine and sparse punctures.

*Metaventrite* finely and sparsely punctured, with a longitudinal depression on posterior seven eighth.

*Abdomen* finely and closely punctured, covered with short golden hairs, with smooth areas laterally on the surface of abdominal segments. The last segment of abdomen covered very large punctures along the outside edge.

*Mesotibia* with outer edge of apex acutely toothed.

*Female genitalia* (Fig. 18) with narrow styli at apex of coxite, and styli rounded apically, covered with setae at apex. Female spermatheca was not found.

##### Distribution.

Known only from the type locality (Australia: Queensland, Edmonton).

##### Remarks.

Micrencaustes (Mimencaustes) serratimaculata is most similar to Micrencaustes (Micrencaustes) gigas MacLeay 1887, due to the similar form and color pattern of body. The new species can be distinguished from it by the mesosternum with coxal lines; very shiny body surface; scutellum not transverse; and the abdomen finely and closely punctured. Micrencaustes (Micrencaustes) gigas is without mesocoxal lines, only moderately shiny, scutellum is transverse, and the ventral surface is sparsely punctured.

##### Etymology.

The species is named for the zigzag mark on pronotum.

## Supplementary Material

XML Treatment for Micrencaustes (Mimencaustes) rotundimaculata

XML Treatment for Micrencaustes (Mimencaustes) serratimaculata
